# Tumor response of temozolomide in combination with morphine in a xenograft model of human glioblastoma

**DOI:** 10.18632/oncotarget.19875

**Published:** 2017-08-03

**Authors:** Anna Lisa Iorio, Martina da Ros, Lorenzo Genitori, Maurizio Lucchesi, Fabiana Colelli, Giacomo Signorino, Francesco Cardile, Giacomo Laffi, Maurizio de Martino, Claudio Pisano, Iacopo Sardi

**Affiliations:** ^1^ Neuro-Oncology Unit, Department of Pediatric Oncology, Meyer Children's Hospital, Florence, Italy; ^2^ BIOGEM Research Institute, Ariano Irpino, Italy; ^3^ Department of Experimental and Clinical Medicine, University of Florence, Florence, Italy

**Keywords:** temozolomide, glioblastoma, morphine, blood-brain barrier, animal model

## Abstract

Despite multimodal treatments comprising, radiation therapy (RT) and chemotherapy with temozolomide (TMZ), the prognosis of glioblastoma multiforme (GBM) remains dismal and consolidated therapy yields a median survival of 14.6 months.

Blood Brain Barrier (BBB) mediated chemoresistance and high dose related toxicity make necessary the development of new therapeutic approach to sensitize GBM to TMZ.

The aim of the present study was to investigate the potential of the treatment morphine *plus* TMZ metronmic doses (1,77 and 0,9 mg/kg) in GBM therapy.

The effect of morphine, on tumor cell growth and P-glycoprothein (P-gp) activity, was investigate in *in vitro* models.

The results demonstrated that GBM cells growth is not influenced by morphine treatment and, for the first time, we show that morphine is an inhibitor of the activity of P-gp efflux transporter who is markedly expressed on BBB.

*In vivo*, response to the treatments TMZ *plus* morphine was investigated in an orthotopic nude mice model of GBM.

Animals treated with TMZ metronomic doses showed a significant tumor growth inhibition compared to untreated mice and association with morphine appears to improve TMZ efficacy.

Moreover, the combination of morphine with lower dose of TMZ result in a cytostatic effect on tumor growth over the period of the pharmacological treatments.

In conclusion this novel approach could be a successful strategy to overcome chemoresistance and side effects TMZ mediated, reducing drug dosage and improving long term response, in GBM therapy.

## INTRODUCTION

GBM is the most common and aggressive primary brain tumor derived from glial progenitors of the central nervous system (CNS) [1, 2].

TMZ, the current standard care for GBM [3, 4], shows chemoresistance shortly after the initiation of treatment [5]; patients median survival is about 12–18 months and only 3 % survive longer than 5 years [6].

Resistance to TMZ has shown to be multifactorial, including changes in the cell cycle, up regulation of mismatch repair genes and MGMT [7] and additional TMZ resistance mechanisms involving P-gp expression, modulation and activity have been recently reported [8, 9, 10].

P-gp belongs to the ATP binding cassette (ABC) transporters and plays the major role in the failure of cancer therapy, limiting the accumulation of a wide range of molecules in numerous tissue including the brain [11].

Different studies have shown that brain concentrations of TMZ are only 17–20 % of the blood levels [12, 13] and TMZ's dose limiting toxicity of leukopenia and thrombocytopenia, with a possible increased risk of opportunistic infections, precludes the use of higher doses which theoretically could result in higher intratumoral concentrations.

It was also reported that antitumor activity of TMZ is highly schedule-dependent [14, 15]; many experimental and preclinical studies suggested that *in vivo* frequent administration of low doses of chemotherapeutic drugs, known as metronomic chemotherapy, could affect tumor endothelium and inhibit tumor angiogenesis, reducing significant side effects [16].

Basing on literature data regarding the pharmacological modulation of BBB by morphine [17], the most commonly used opioid in oncological patients, we have recently demonstrated that this agent is able to increase delivery and efficacy of doxorubicin (Dox) in animal models, without increasing systemic toxicities [18, 19, 20, 21].

However, results about the effect of morphine on tumor cells are numerous and often controversial.

Some authors reported that morphine can reduce the efficacy of chemotherapy, stimulating tumor cell growth in brain, breast and renal cancer [22, 23, 24, 25]. Other reported that the association of morphine with chemotherapeutic agents result in an improved antitumor effect and suppression of cancer cells proliferation [26, 27]

Considering all this findings, the aim of the present study was further investigate the effect of morphine on tumor cells and explore the potential of combined treatments morphine *plus* TMZ in GBM therapy, in order to overcome chemoresistance and side effects TMZ mediated.

## RESULTS

### Effect of morphine on GBM cells proliferation

We tested the modulatory action of morphine on tumor cells growth treating two human GBM cell lines (U87MG and A172) with different doses of morphine (10, 20, 40 μM) for 24 and 48 hours.

**Figure 1:**
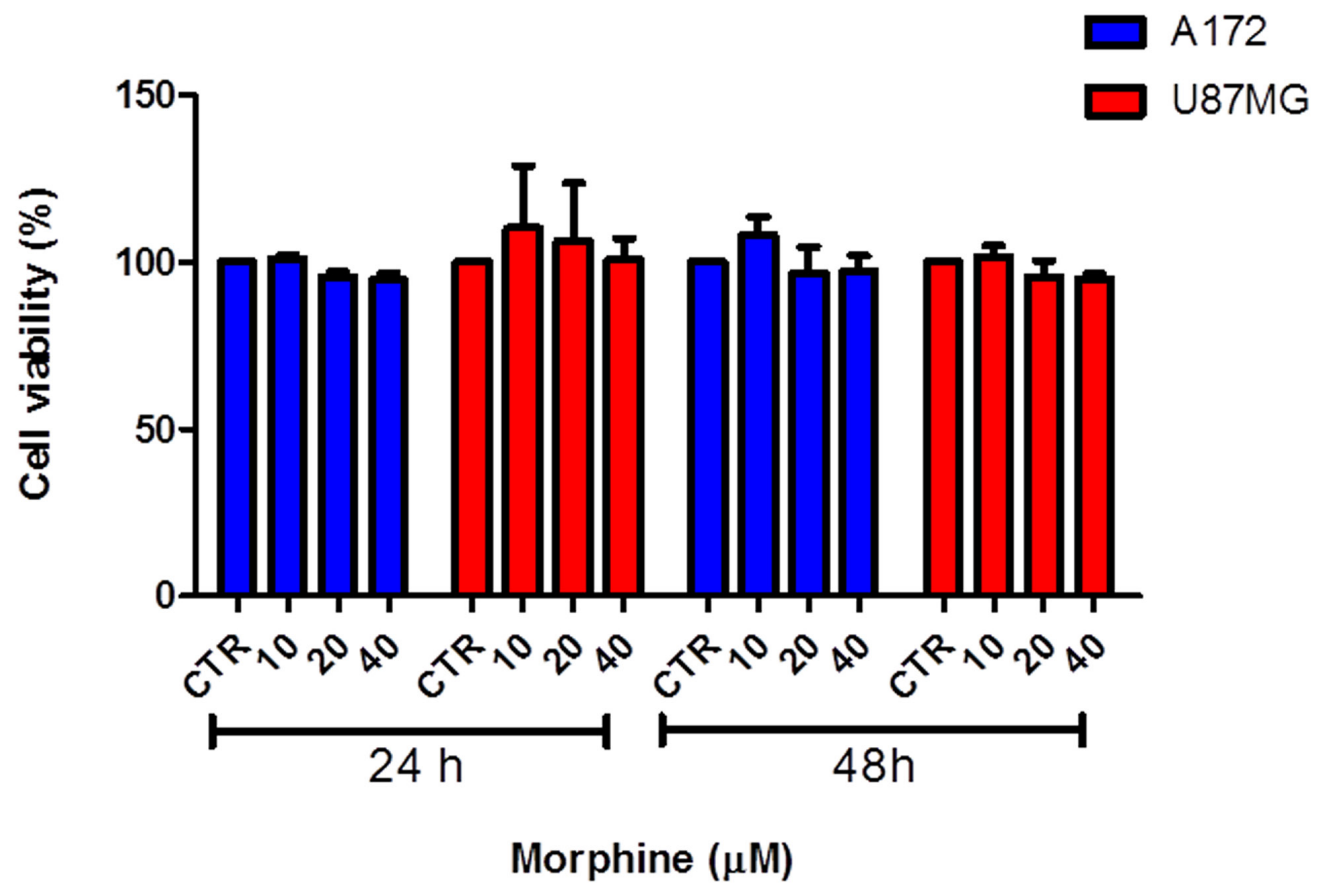
Effect of morphine on human GBM cells growth U87MG and A172 cells were treated with various concentrations of morphine for 24 and 48 hours. No changes in cell growth can be observed comparing controls *vs* all morphine dosages, in both cell lines and times. Data were expressed as mean ± SD.

As show in Figure 1, no difference in cell proliferation, at 24 hours, can be observed comparing control groups and morphine treated groups, in both cell lines. Data were confirmed comparing treated groups *vs* control groups after 48 hours of treatment, in both cell lines.

### Effect of morphine on ATPase activity of P-gp

The drug efflux function of P-gp is coupled to ATP hydrolysis, which is stimulated in the presence of P-gp substrates.

**Figure 2:**
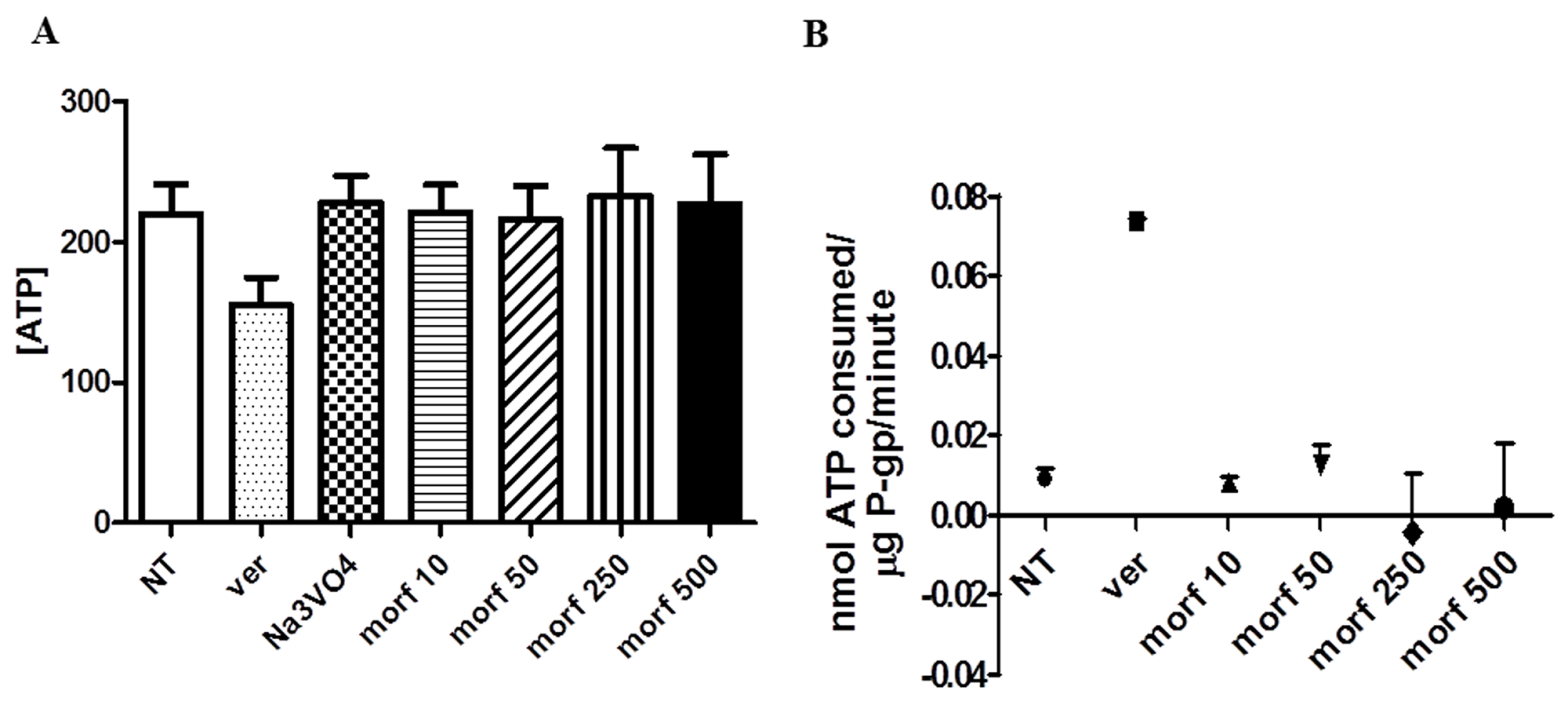
Interaction between morphine and P-gp ATPase activity **(A)** After 40 min of incubation with various concentrations of morphine, the remaining unmetabolized ATP was detected as a luciferase-generated luminescent signal. Statistical differences can be observed comparing ATP concentration of Verapamil *vs* all doses of TC (^*^p<0,01). Data were expressed as mean ± SD. **(B)** ATP consumption reflected P-gp ATPase activity. In presence of morphine the function of the efflux transporter was lower than NT and Verapamil, indicating that morphine is a functional inhibitor of P-gp. Data were expressed as mean ± SD.

Our data indicated that the ATP concentration measured in samples treated with different concentration of morphine are significantly higher compared to Verapamil samples (^*^p< 0,01) (Figure 2A), indicating that in presence of morphine there was a reduced consumption of ATP.

To assess the effect of morphine on the P-gp ATPase activity, P-gp-mediated ATP hydrolysis was measured, with the appropriate formula reported in materials and method section, using ATP concentration of morphine and sodium orthovanadate samples. As shown in Figure 2B, all morphine doses are able to reduce the ATPase activity of P-gp.

### Effect of combined treatment TMZ plus morphine on tumor growth in glioblastoma xenograft

Basing on literature data reporting that TMZ metronomic administrations are more effective than a single bolus dose [14], a preliminary experiment was realized to evaluate TMZ concentration for treatments.

Animals were treated daily with TMZ (1,77-2,66 and 4 mg/kg) for five weeks and tumor growth was monitored trough weekly BLI acquisition.

**Figure 3:**
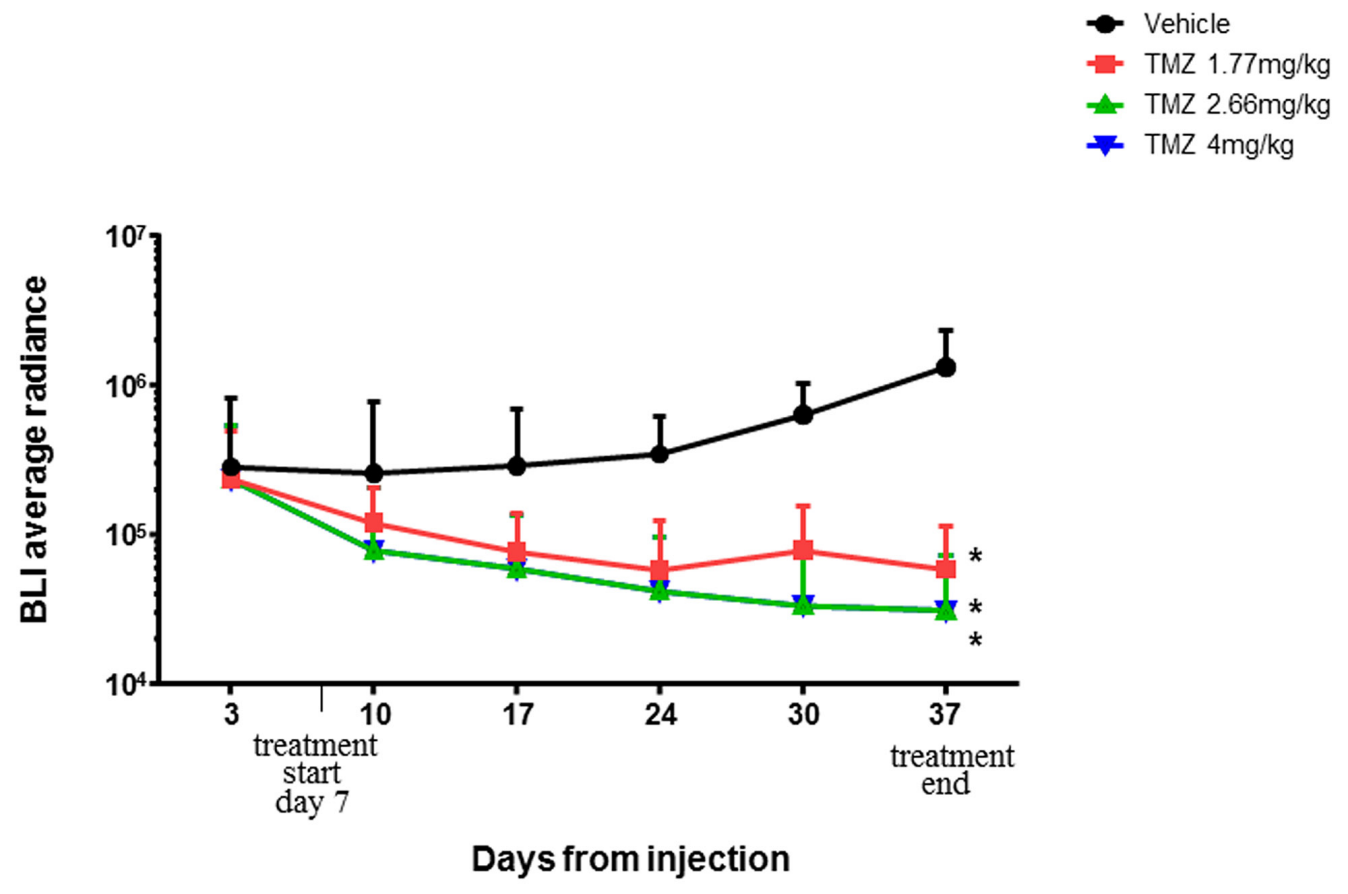
BLI average radiance throughout the experimental time Statistical significant differences were observed comparing BLI Averageradiance of control group *vs* all TMZ treated groups (p<0,05). Physiological solution 5 ml/kg (violet line), TMZ 1,77 mg/kg (blue line), TMZ 2,66 mg/kg (red line), and TMZ 4 mg/kg (green line). Data were expressed as mean ± SD.

Data shown in Figure 3 revealed that all TMZ doses were effective in tumor growth inhibition compared to control (p<0,05) so 1,77 mg/kg, the lowest concentration that achieved a significant results, were chosen as dosage for next experiments.

The first step experiment was then performed combining TMZ treatment (1,77 mg/kg) with morphine (10 mg/kg) [16–20].

**Figure 4:**
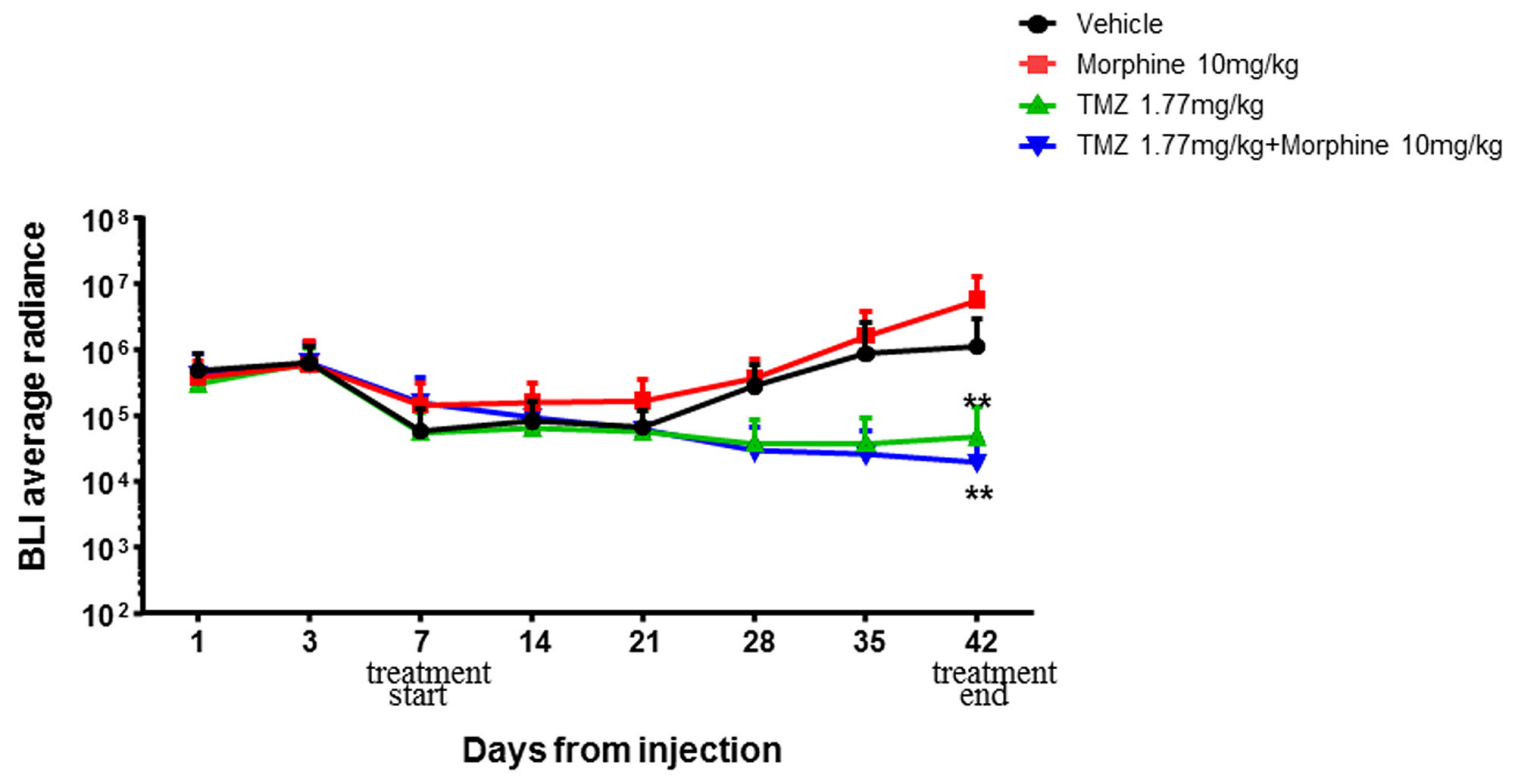
BLI average radiance curves distinctly for group of treatment and days of treatment Atday +42, morphine treatment (red) is not associated with an increased tumor growth compared to control group (black) while statistically significant differences (^**^p<0,01) were found between vehicle group *vs* TMZ 1,77 mg/kg group (green) and TMZ 1,77 mg/kg plus morphine 10 mg/kg group (blue). Data were expressed as mean ± SD.

One week after tumor implantation, animals were divided in four groups and treated following the first step experiment's schedule as described in materials and methods section. Time-course and bioluminescence (BLI) evaluation of tumor growth are reported in Figure 4.

**Figure 5:**
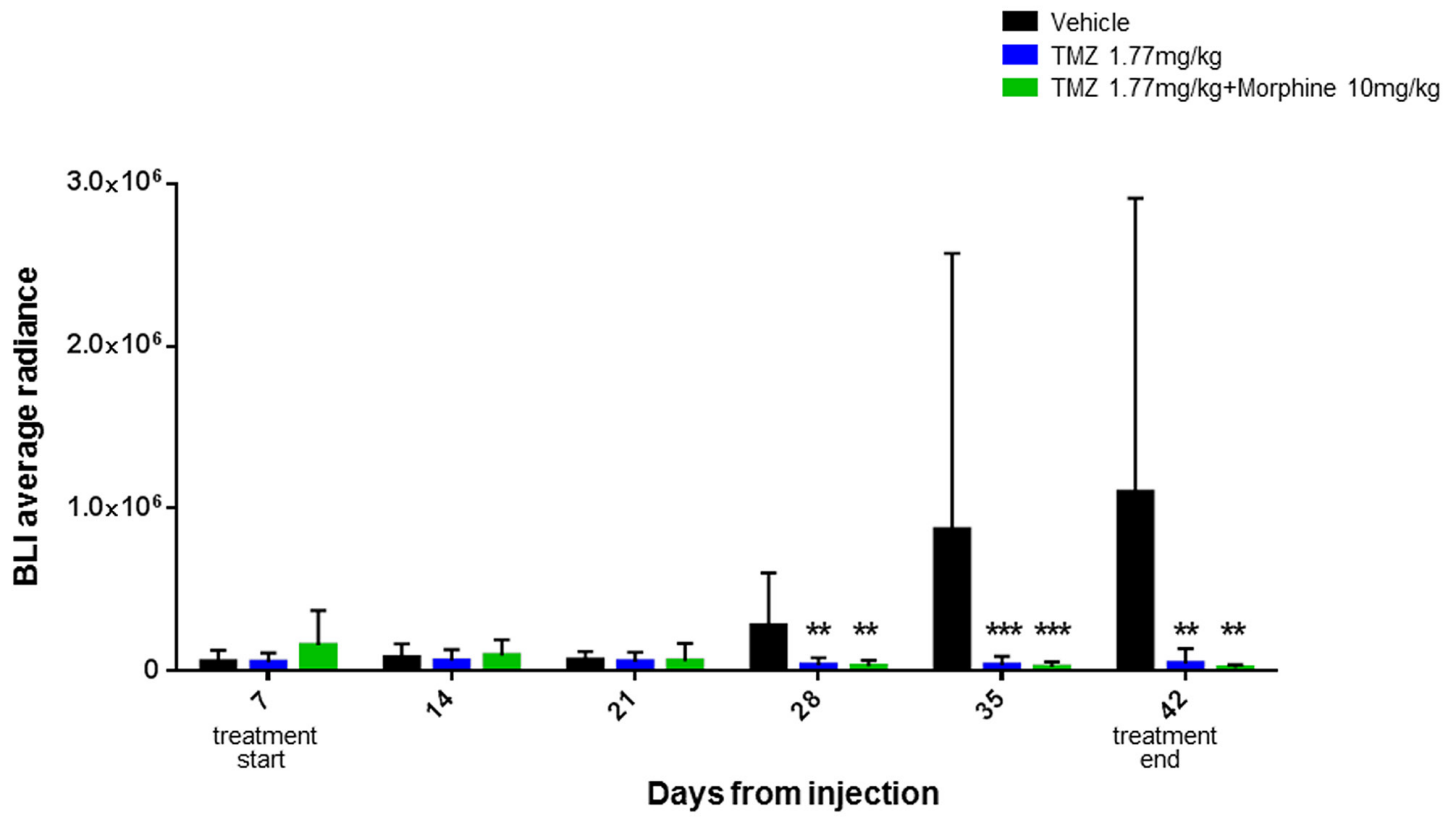
Therapeutically effect of TMZ metronomic treatments TMZ 1,77 mg/kg, whit or without morphine, is effective in tumor growth inhibition from early administration and not only at the end of the experiment as cumulative doses effect. Statistical analysis wasperformed, at day +28,+35 and +42, between vehicle group (black), TMZ 1,77 mg/kg group (green) and TMZ 1,77 mg/kg plus morphine 10 mg/kg group (blue) (^**^p<0,01, ^***^p<0,001). Data were expressed as mean ± SD.

Statistical significant results were obtained comparing BLI data of treated groups *vs* control group, at days +28, +35 and +42. At day +28 the BLI Average Radiance of control group was 2,81E+05 *vs* 3,67E+04 of TMZ group (^**^p<0,01) and 2,97E+04 of TMZ *plus* morphine group (^**^p<0,01). BLI Average Radiance of control group, at day +35, was 8,71E+05 *vs* 3,68E+04 of TMZ group (^***^p<0,001) and 2,58E+04 of TMZ *plus* morphine group (^***^p<0,001). On the last BLI acquisition, day +42, the Average Radiance of control group was 1,10E+06 *vs* 4,70E+04 of TMZ group (^**^p<0,01) and 1,95E+04 of TMZ *plus* morphine group (^**^p<0,01) (Figure 5). At day +42 two animals of control group died before BLI acquisition.

**Figure 6:**
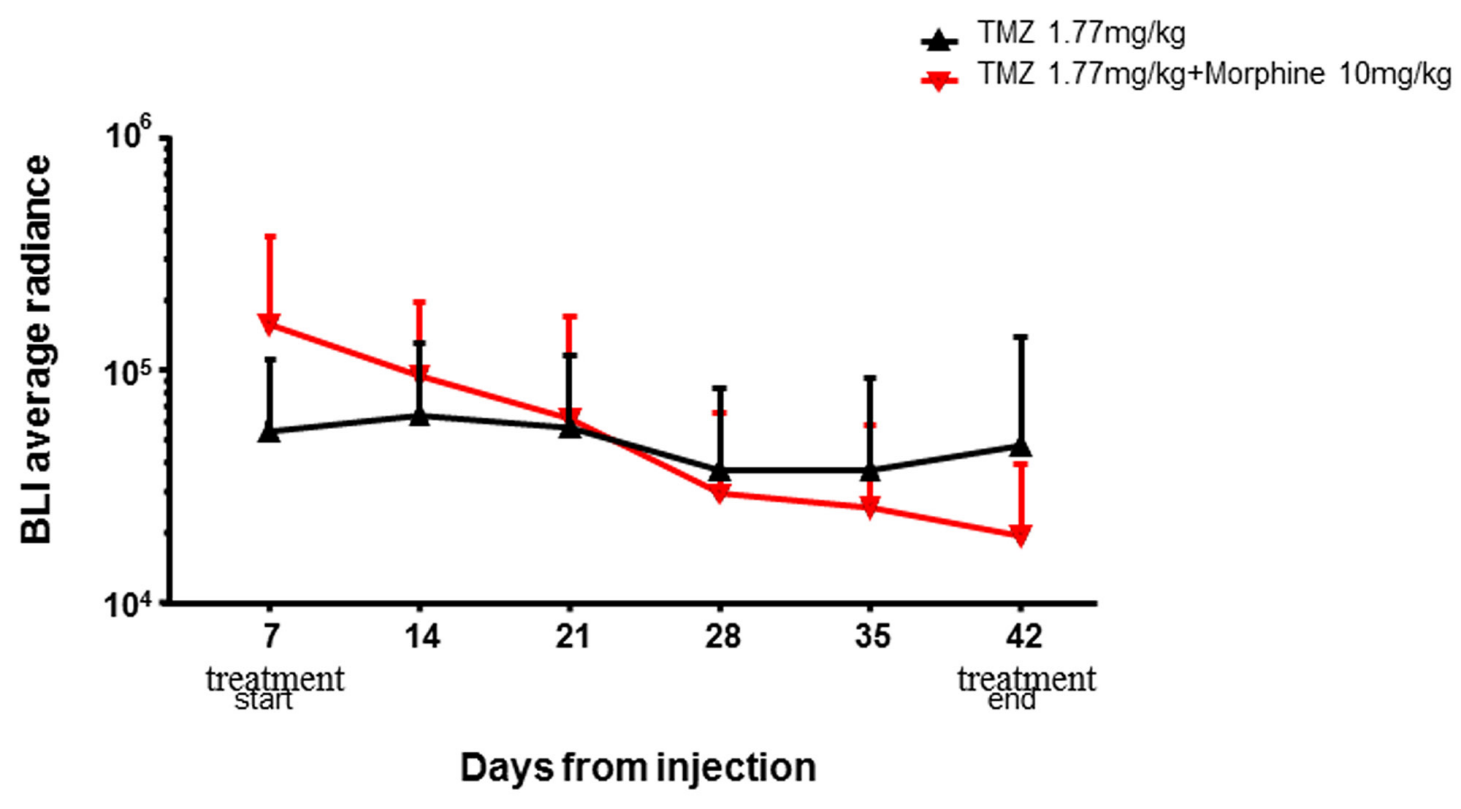
BLI average radiance curves distinctly for groups and days of treatment TMZ (1,77 mg/kg) shows an important effect in combination with morphine (10 mg/kg), determining a BLI reduction of 2,5 fold compared to BLI measured in group treated with only TMZ (1,77 mg/kg). Data were expressed as mean ± SD.

**Figure 7:**
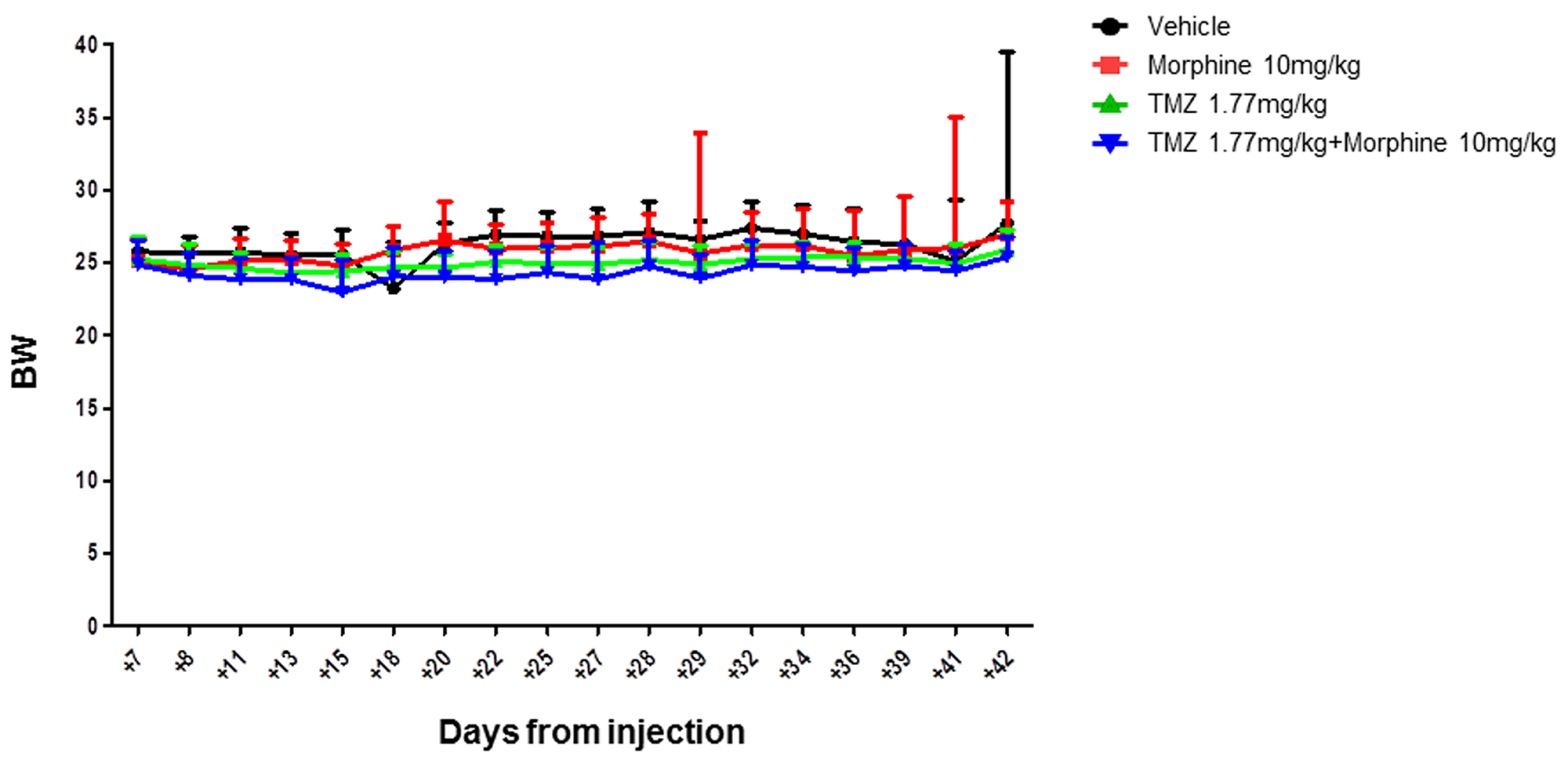
Body weight evaluation throughout the experimental time Side effects of treatments were evaluated in terms of body weight variation. No statistical significant differences were reported between all different groups, indicating that the improved antitumor activity of TMZ *plus* morphine treatment was not associate to an increased systemic toxicity. Data were expressed as mean ± SD.

This data indicated that TMZ metronomic treatments have significant effect, from day +28 to day +42, on tumor growth but, analyzing the global trends of tumor regression, the BLI Average Radiance of TMZ *plus* morphine group was 2.5 fold lower than value observed in TMZ group (Figure 6). No differences in body weight loss were observed comparing control group *vs* all treated groups (Figure 7).

### Effect of morphine in combination with lower dose of TMZ

We also investigated if combined treatment with morphine could make possible further reduction of TMZ dose, preserving the therapeutically effect of the alkylating agent.

To test this hypothesis, the second step experiment was performed comparing the effect of an half dose of TMZ (0,9 mg/kg), with or without morphine, *vs* TMZ (1,77 mg/kg).

One week after tumor implantation, animals were divided in four groups and treated following the second step experiment's schedule as described in materials and methods section.

Statistical significant results were obtained comparing BLI data of treated groups and control group at the end of the treatments (day +42).

**Figure 8:**
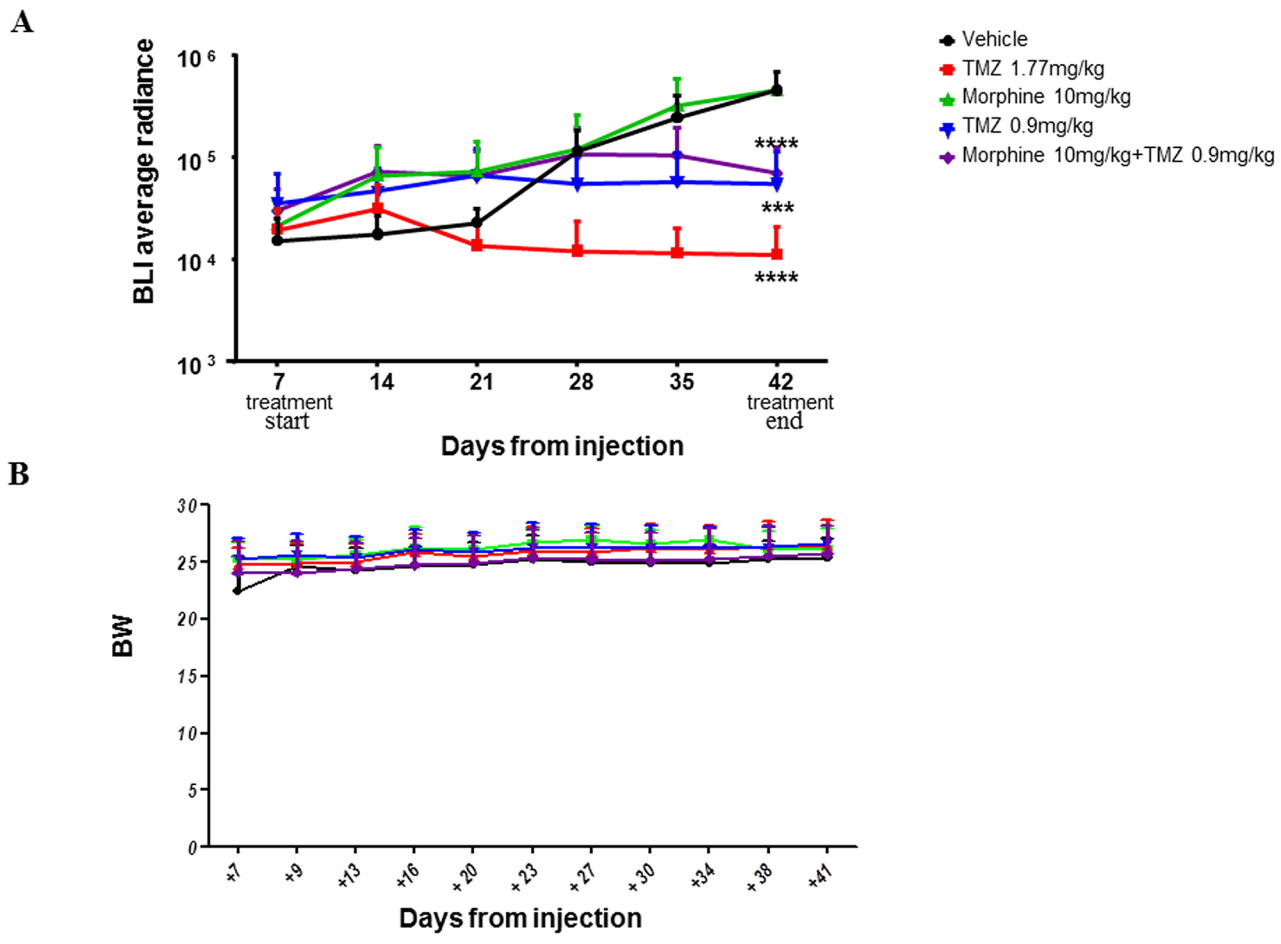
BLI signals throughout the experimental time **(A)** Statistical analysis was performed between vehicle and the other experimental groups (^****^p<0,0001; ^***^p<0,001). Data were expressed as mean ± SD. **(B)** Side effects of treatments were evaluated in terms of body weight variation. No statistical significant difference were reported between all different groups. Data were expressed as mean ± SD.

The BLI Average Radiance of control group was 4,55E+05 *vs* 1,12E+04 of TMZ (1,77 mg/kg) group (^****^p<0,0001), 5,51E+04 of TMZ (0,9 mg/kg) group (^***^p<0,001) and 6,98E+04 of TMZ (0,9 mg/kg) *plus* morphine group (^****^p<0,0001) (Figure 8A).

At the end of the treatments (day +42), TMZ (1.77 mg/kg) determined a tumor volume inhibition (TVI%, reported as BLI reduction and calculated comparing BLI value with BLI of control group treated with vehicle only) of 97,5%; meanwhile, TMZ (0,9 mg/kg), alone or in combination with morphine, determined a TVI between 84% (TMZ 0,9 mg/kg *plus* morphine) and 88% (TMZ 0,9 mg/kg), without significant difference between the two groups. No differences in body weight loss were observed comparing control group *vs* all treated groups (Figure 8B).

### Combined treatment with morphine increases long-term response to TMZ

**Figure 9:**
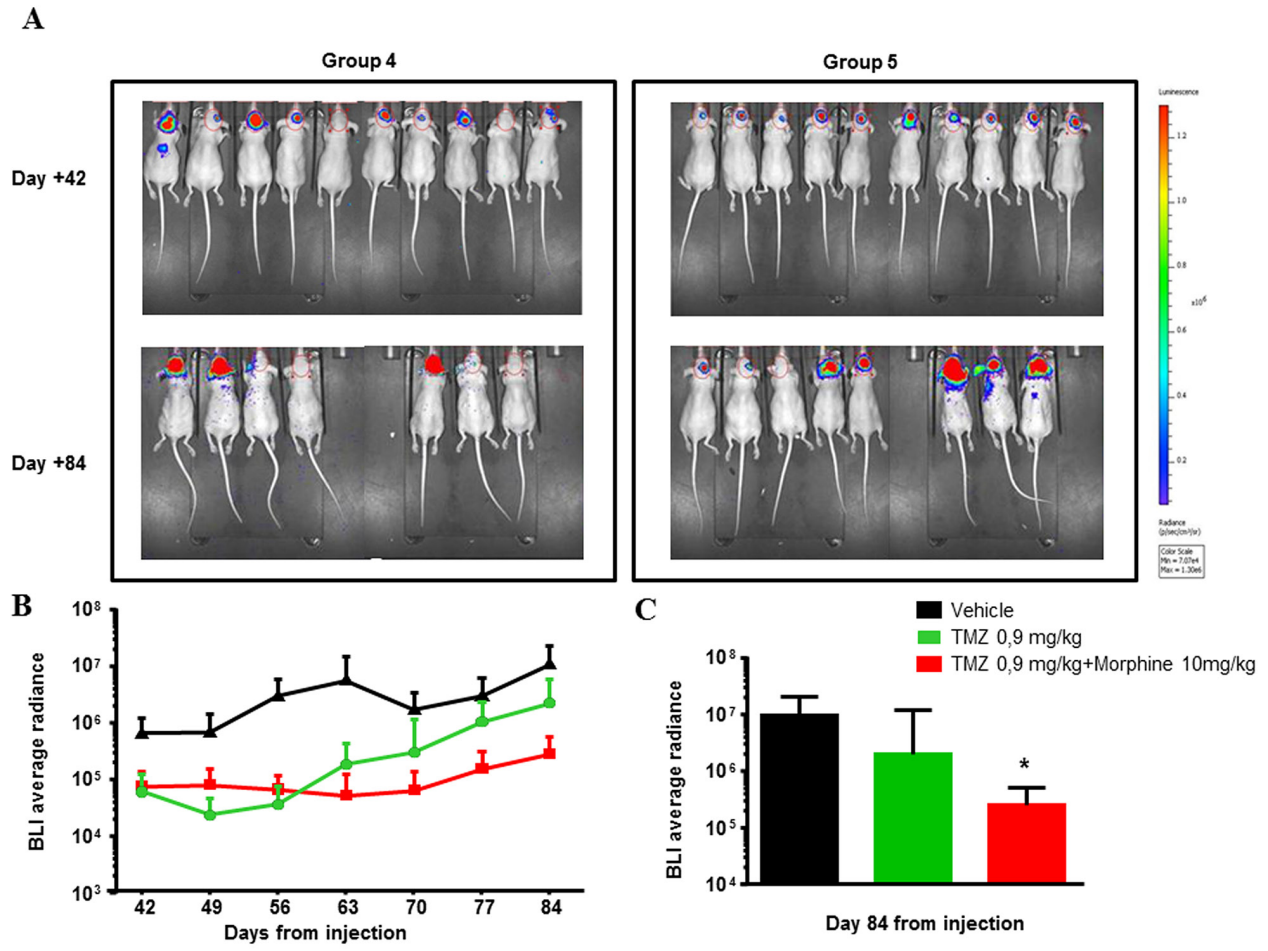
Long-term response to TMZ **(A)** Images of BLI acquisition of Group 4 (TZM 0,9 mg/kg) and Group 5 (TZM 0,9 mg/kg *plus* morphine) of the Second Step Experiment, at day +42 (end of treatment) and day+ 84 (sacrifice). BLI is expressed as a total radiance in photons per sec/cm^2^ per steradian. The colorimetric scale represents the range of radiance values (red=highest value; blu=lowest value) which translates to tumor growth. **(B)** BLI Average radiance curves distinctly for group of treatment and days of treatment. TMZ (0,9 mg/kg) shows an important effect in combination with morphine (10 mg/kg), determining a BLI reduction of 7,8 fold compared to BLI measured in group treated with only TMZ (0,9 mg/kg). Data were expressed as mean ± SD. **(C)** Comparison of TMZ (0,9 mg/kg) alone or in combination with morphine (10 mg/kg) at day +84 *vs* vehicle, showed a significant reduction of BLI of combination TMZ (0,9 mg/kg) *plus* morphine (10 mg/kg) (^*^p<0,05) while TMZ (0,9 mg/kg) is not statistically significant. Data were expressed as mean ± SD.

Long-term response to the treatments was evaluated from day +42, end of treatments, until day +84 (all animals were sacrified) (Figure 9A).

At the end of the treatments, TMZ (1.77 mg/kg) showed a TVI of 97,5%; this inhibition persists for the whole experimental period and reaches up to over 100% (day +84).

The combination TMZ 0,9 mg/kg *plus* morphine maintained a more or less stable reduction after the end of the treatments until the day +84 with a TVI of 97,3% while, the effect of TMZ 0.9 mg/kg alone ended after the end of treatments (TVI from 88% to 79%, day +42 and +84 respectively). In accordance, the BLI Average Radiance of TMZ 0,9 mg/kg *plus* morphine group was 7.8 fold lower than value measured in group treated with only TMZ 0.9 mg/kg (Figure 9B) and, at day +84, statistical significant difference were observed comparing BLI data of co-treated group *vs* control group (^*^p<0,05) (Figure 9C).

As control, animals were treated with morphine alone; in this case morphine administration does not determine any tumor volume variation compared to animals treated with the vehicle at the end of the treatments (day +42). Conversely, we found a 90,7% of TVI at day +84, but we have to underline that this effect could be due to the lower number of animals survived in this group.

## DISCUSSION

Currently adjuvant treatment of GBM is based on the association of RT and TMZ administrations [3].

However, chemoresistance and TMZ-induced side effects (anemia, lymphopenia, neutropenia, severe thrombocytopenia [3, 28, 29, 30] and liver injury [31, 32]) represent a big challenge in the management of oncological patients.

The development of combination therapy that could sensitize GBM to TMZ is essential so, the aim of the present study was investigate the potential of an innovative combinational approach with morphine to improve chemotherapy effectiveness, reducing drug dosage and consequently TMZ-induced side effects.

Morphine is the most used drug for the pain management in oncological patients but, literature data regarding the effect of the opioid agent on tumor cells growth are numerous and controversial [22, 23, 24, 25, 26]

On the other hand, *in vivo* studies have suggested that stress associated with high doses morphine administration or withdrawal can influence the BBB function [17, 33, 34] and allow the accumulation and the efficacy of anticancer drugs [21].

Judging from all these facts, we first investigated the effect of morphine treatments on GBM tumor cells growth. Our *in vitro* results showed that exposure to micromolar concentrations of morphine not influenced cells growth (Figure 1), indicating that this agent don't represent a proliferative stimulus for the tested GBM cells.

To well understand the molecular mechanism underlying the effect of morphine on BBB permeability, we have further investigated the possible interaction between the opioid agent and the efflux protein P-gp, overexpressed in the endothelial cells of the BBB and involved in resistance to many chemotherapeutic drugs.

For the first time, our *in vitro* results demonstrated that morphine is an effective P-gp inibhitor (Figure 2B), suggesting that the opioid agent could enhances the effects of chemotherapeutic drugs in brain tissue through reduction of the ATP binding cassette transporter function.

Based on the *in vitro* data acquired in this study and experimental and clinical literature suggesting a key role for P-gp in GBM resistance to TMZ [8, 9], we determined to evaluate the efficacy of combination of morphine with TMZ metronomic treatment in an *in vivo* model of GBM.

First step experiment data have indicated that morphine treatment is not associated with an increased tumor growth compared to control group (Figure 4) and also that TMZ 1.77mg/kg metronomic treatment, with or without morphine, is effective in tumor growth inhibition from early administrations and not only at the end of the experiment as cumulative doses effect (Figure 5).

However, analyzing the global trend of tumor regression it was possible to appreciate the important effect that TMZ showed in combination with morphine. At day +42, the BLI Average Radiance of TMZ *plus* morphine group was 2.5 fold lower than value measured in group treated with only TMZ (Figure 6). Moreover, this improvement in antitumor activity of TMZ *plus* morphine treatment was not associate to an increased systemic toxicity, evaluated as difference in body weight loss between co-treated group and all other groups (Figure 7).

This data encouraged us to test if the combination with morphine could make possible a further reduction of TMZ dosage, with additional benefits in terms of side effects.

Co-treatment with TMZ (1,77 and 0,9 mg/kg), with or without morphine, determined an important tumor growth inhibition, TMZ (1,77 mg/kg) showed a TVI of 97,5% meanwhile, TMZ (0,9 mg/kg), alone or in combination with morphine, determined a tumor reduction between 84% and 88%.

Moreover, from day +42 until day +84, we observed that while the effect of the half dose of TMZ alone ended after the end of treatments, the combination of half dose of TMZ *plus* morphine maintained a more or less stable reduction from the end of the treatments until the day +84, with a TVI of 97,3%.

As reported in Figure 9B, BLI values of TMZ 0.9 mg/kg, with or without morphine, started from the same point (day+42) but, while BLI values of TMZ 0.9 mg/kg group increased, the BLI values of TMZ 0.9 mg/kg *plus* morphine group remained stable over the time reaching, at day +84, a BLI fold decrease of 7.8 compared to TMZ 0.9 mg/kg group.

The cytostatic effect of the co-treatment was further supported comparing BLI values of this group *vs* control group. In Figure 9B can be observed that both treatments curves are below the control but, at the end of the experiment, there was a statistical significant difference (^*^p<0,05) in tumor volumes only between control and TMZ 0.9 mg/kg *plus* morphine groups (Figure 9C).

In conclusion, our results demonstrated that morphine is a P-gp inhibitor, TMZ metronomic treatment are effective in GBM therapy and also that combination TMZ *plus* morphine shows an increased effectiveness in tumor growth inhibition and long term response in a GBM xenograft model.

More study are necessary to well understand the appropriate schedule of co-treatment but, this safe and non-invasive approach could be a successful strategy to overcome chemoresistance and side effects TMZ mediated.

## MATERIALS AND METHODS

### Ethics statement

Investigation has been conducted in accordance with the ethical standards and according to the Declaration of Helsinki and according to national and international guidelines and has been approved by the authors’ institutional review board.

### Drugs and reagents

TMZ (Sigma Aldrich) dissolved in sterile H_2_O was administered, at different concentrations, *per os* (p.o.) in a volume of 5 ml/kg. Morphine (Molteni & C s.p.a.) 10 mg/kg was dissolved in sterile saline and subcutaneously (sc) administered in a volume of 5 ml/kg. All chemicals and solvents were of the highest purity available from commercial sources and used without further purification.

### MTT assay

The effect of morphine on tumor cells growth was measured using the MTT assay (*in vitro* toxicology assay kit MTT based, Sigma). The key component of this assay is (3-[4,5-dimethylthiazol-2yl]-2,5-diphenyl tetrazolium bromide), a yellow salt that mitochondrial dehydrogenases of viable cells convert into purple formazan crystals, whose concentration is measured spectrophotometrically. We have conducted preliminary experiments to determine the best seeding concentration for U87MG and A172 cells. Consequently, cells were seeded at the following densities: A172, 6×10^4^ cells/well and U87MG, 4×10^4^ cells/well in 24-well plates. After 24 hours, the cells were treated morphine 10, 20 and 40 μM for 24 and 48 hours. The MTT assay was performed following the manufacturer's instructions. The plates were placed on a shaker for 10 minutes to enhance solubilization of the precipitate. The absorbance of each well was then measured on a MULTISKAN FC (Thermo Scientific) microplate reader at a test wavelength of 550 nm. All experiments were performed tree times in triplicate

### Adenosine triphosphatase (ATPase) assay of P-gp

The ATPase activities of P-gp were determined using the luminescent ATP detection kit (Pgp-Glo Assay Systems, Promega, Madison, WI) according to the manufacturer's instructions. Briefly, 1.25 mg/mL P-gp membranes and 25 mM MgATP were incubated in absence or presence of morphine, at 37 °C for 40 minutes. The relative lights units (RLU), representing the luciferase-generated luminescent signal, were detected on a plate-reading luminometer (The GloMax® 96 Microplate Luminometer, Promega).

RLU of each sample was interpolate with RLU of ATP standards to obtain ATP concentrations in control (NT) and test compounds (TC). The impact of TC on P-gp ATPase activity was examined comparing NT and samples treated with different concentrations of morphine to Na_3_VO_4_ (sodium orthovanadate)-treated control and Verapamil-treated control, that are respectively an inhibitor and a stimulator of the P-gp activity.

Basal P-gp ATPase activities were determined as the difference between the ATP hydrolysis in presence or absence of Na_3_VO_4_,

$$\frac{\left([{\text{ATP}}_{\text{Na3VO4}}]-[{\text{ATP}}_{\text{NT}}]\right)}{\left(\text{25}\mu \text{g P}-\text{gp x 4}0\text{ minutes}\right)}$$

Verapamil and morphine-stimulated P-gp ATPase activity was measured in presence of varying concentrations of morphine,

$$\frac{\left([{\text{ATP}}_{\text{Na3VO4}}]-[{\text{ATP}}_{\text{TC}}]\right)}{\left(\text{25}\mu \text{g P}-\text{gp x 4}0\text{ minutes}\right)}$$

All experiments were performed two times in triplicate.

### Animals

A total of 136 Foxn1 nude mice have been used in this study. The procedure of animals accommodation and care have been described in detail previously [21].

Briefly, 6- to 7-weeks-old nude female mice Foxn1, weighing approximately 21-28 g, were housed inside polysulfone cages (4-5 mice/cage) with stainless steel cover-feed and sterilized and dust-free bedding cobs. Mice were maintained in cages with paper filter covers; food and bedding were sterilized. All manipulations were carried out according to the European Community guidelines for animal care (DL 116/92, application of the European Communities Council Directive 86/609/EEC). Animals were inspected throughout experiments for mortality. Physical appearance, behaviour, general and local clinical signs were also observed; all efforts were made to minimize animal sufferings and mice showing clinical signs of pain and distress were sacrificed for humane reasons by CO_2_ inhalation. At the end of experiments, all mice were sacrificed by CO_2_ inhalation.

### Tumor implantation

U87MG-luc2 (PerkinElmer Italia S.P.A., Monza, Italy), a human GBM cell line stably transfected with firefly luciferase gene (luc2), was used to establish the orthotopic glioma model.

Cells were cultured in Eagle's Minimum Essential Medium containing heat-inactivated FBS at final concentration 10% and maintained in a humidified atmosphere of 5% CO_2_-95% air at 37°C. On the day of the tumor implantation mice were micro-injected with 3×10^5^ U87MG-luc2 cells, suspended in 5 μl of saline solution, in the left lobe of brain with infusion of 1 μl/min (with Hamilton syringe).

Following intracranial tumor injection, BLI acquisitions were performed, at day 0, 3 and 7, for baseline data.

### Experimental design

After tumor implantation (day +7), mice were sorted on the basis of BLI Average Radiance and randomly allocated as follows.

### Pilot experiment

Group 1 (Ctr): naïve mice received physiological solution (5 ml/kg). Group 2 (TMZ 1,77 mg/kg): daily administrations from day 7 to 37. Group 3 (TMZ 2,66 mg/kg): daily administrations from day 7 to 37. Group 4 (TMZ 4 mg/kg): daily administrations from day 7 to 37.

Each experimental group contained 7 animals. Tumor growth and weight were carried out weekly and biweekly respectively, for five weeks.

### First step experiment

Group 1 (Ctr): naïve mice received physiological solution (5 ml/kg).

Group 2 (morphine): morphine weekly administrations (days 7, 14, 21, 28, 35).

Group 3 (TMZ 1,77mg/kg): TMZ daily administrations from day 7 to 42.

Group 4 (TMZ 1,77 mg/kg+morphine): weekly administrations of morphine and daily administrations of TMZ.

Each experimental group contained 10 animals. Tumor growth and weight were carried out weekly and biweekly respectively, for five weeks.

### Second step experiment

Group 1 (Ctr): naïve mice received physiological solution (5 ml/kg).

Group 2 (morphine): morphine weekly administrations (days 7, 14, 21, 28, 35). Group 3 (TMZ 1,77 mg/kg): TMZ daily administrations from day 7 to 42.

Group 4 (TMZ 0,9 mg/kg): daily administrations of TMZ from day 7 to 42.

Group 5 (TMZ 0,9 mg/kg+morphine): weekly administrations of morphine and daily administrations of TMZ.

Each experimental group contained 10 animals. Tumor growth and weight were carried out weekly and biweekly respectively, for five weeks. Animals of each groups were observed from day +42 (end of treatments) until the day +84 for long-term response evaluation.

### Bioluminescence acquisition

Tumor growth and response to the treatments were monitored by BLI acquisitions, using IVIS 200 Spectrum Imaging System (PerkinElmer). BLI acquisitions were performed at day 0, 3, 7 and then weekly until the end of experiments.

In detail, mice were i.p. administered with 150 mg/kg/10 mL of Dluciferin (PerkinElmer, Xenolight D-Luciferin Potassium Salt, 1g, #122799, batch K9906PE, stored at -20°C) and, 30 minutes upon luciferin administration, were anesthetized by gas anesthesia (3% isoflurane for induction, 1.5% for maintaining). All animals were placed into the IVIS 200 Imaging System to be imaged. BLI was expressed as Average Radiance in photons per sec/cm^2^ per steradian.

### Body weight

Body weight (BW) variation was calculated as follows: [(actual BW (g) x 100) / initial BW (g)] – 100. Measurements were carried out biweekly and, as humane endpoint, animals showing a BW reduction ≥ 15% were sacrificed by CO_2_ inhalation.

### Statistical analysis

Data were expressed as mean ± SD. Statistical analyses were performed using U-Test between two groups while comparison between multiple groups were performed using 1 or 2 way ANOVA followed by post hoc corrections, as appropriate. All analyses were done using GraphPad Prism 5 and P<0,05 was considered statistically significant.
